# Diagnostic Efficacy of ^123^Iodo-Metaiodobenzylguanidine SPECT/CT in Cardiac vs. Neurological Diseases: A Comparative Study of Arrhythmogenic Right Ventricular Cardiomyopathy and α-Synucleinopathies

**DOI:** 10.3390/diagnostics15010024

**Published:** 2024-12-26

**Authors:** Johannes M. Hagen, Maximilian Scheifele, Mathias J. Zacherl, Sabrina Katzdobler, Alexander Bernhardt, Matthias Brendel, Johannes Levin, Günter U. Höglinger, Sebastian Clauß, Stefan Kääb, Andrei Todica, Guido Boening, Maximilian Fischer

**Affiliations:** 1 Department of Nuclear Medicine, Ludwig-Maximilians-University, 81377 Munich, Germany; j.hagen@med.uni-muenchen.de (J.M.H.); maximilian.scheifele@med.uni-muenchen.de (M.S.); mathias.zacherl@med.uni-muenchen.de (M.J.Z.); matthias.brendel@med.uni-muenchen.de (M.B.); andrei.todica@med.uni-muenchen.de (A.T.); guido.boening@med.uni-muenchen.de (G.B.); 2 Department of Neurology, Ludwig-Maximilians-University, 81377 Munich, Germany; sabrina.katzdobler@med.uni-muenchen.de (S.K.); alexander.bernhardt@med.uni-muenchen.de (A.B.); johannes.levin@med.uni-muenchen.de (J.L.); guenter.hoeglinger@med.uni-muenchen.de (G.U.H.); 3 German Center for Neurodegenerative Diseases (DZNE), 81377 Munich, Germany; 4 Munich Cluster for Systems Neurology (SyNergy), 81377 Munich, Germany; 5 Interfaculty Center for Endocrine and Cardiovascular Disease Network Modelling and Clinical Transfer (ICONLMU), LMU Munich, 81377 Munich, Germany; sebastian.clauss@med.uni-muenchen.de (S.C.); stefan.kaab@med.uni-muenchen.de (S.K.); 6 Department of Medicine I, University Hospital Munich, Ludwig-Maximilians-University, 81377 Munich, Germany; 7 DZHK (German Centre for Cardiovascular Research), Partner Site Munich Heart Alliance, 81377 Munich, Germany; 8 Institute of Surgical Research, Walter-Brendel-Center of Experimental Medicine, University Hospital, LMU Munich, 81377 Munich, Germany; 9 European Reference Network for Rare, Low Prevalence and Complex Diseases of the Heart (ERN GUARD-Heart), 81377 Munich, Germany

**Keywords:** ARVC, α-synucleinopathies, ^123^I-MIBG, SUV, heart, RV

## Abstract

**Background/Objectives**: ^123^Iodo-metaiodobenzylguanidine single photon emission computed tomography/computed tomography (^123^I-MIBG SPECT/CT) is used to evaluate the cardiac sympathetic nervous system in cardiac diseases such as arrhythmogenic right ventricular cardiomyopathy (ARVC) and α-synucleinopathies such as Parkinson’s diseases. A common feature of these diseases is denervation. We aimed to compare quantitative and semi-quantitative cardiac sympathetic innervation using ^123^I-MIBG imaging of ARVC and α-synucleinopathies. **Methods**: Cardiac innervation was assessed using ^123^I-MIBG SPECT/CT in 20 patients diagnosed with definite ARVC and 8 patients with clinically diagnosed α-synucleinopathies. Heart-to-mediastinum-ratio (H/M-ratio), as semi-quantitative, was evaluated. Additionally, standardized uptake value (SUV), as quantitative, was measured as SUV_median_, SUV_max_, and SUV_peak_ in the left ventricle (LV), the right ventricle (RV), and in the global heart, based on a CT scan following quantitative image reconstruction. **Results**: The quantification of ^123^I-MIBG uptake in the LV, the RV, and the global heart was feasible in patients suffering from α-synucleinopathies. SUV_median_, and SUV_peak_ demonstrated a significant difference between ARVC and α-synucleinopathies across all regions, with the α-synucleinopathy group showing a lower uptake. In addition, the H/M ratio showed significantly lower uptake in patients with α-synucleinopathies than in patients with ARVC. **Conclusions**: Patients with α-synucleinopathies demonstrate significantly lower cardiac innervation in semi-quantitative and quantitative examinations than ARVC patients. The comparison of semi-quantitative and quantitative examinations suggests that quantitative examination offers an advantage. Quantitative analysis can be performed separately for the LV, RV, and global heart. However, analyzing the LV or RV does not provide additional benefit over analyzing the global heart in distinguishing between α-synucleinopathies and ARVC. Considering the different clinical manifestations of these two diseases, the absolute SUV values should not be generalized across different pathologies, and disease-specific ranges should be used instead.

## 1. Introduction

Several methods are available in nuclear medicine to investigate pathophysiological cardiac processes: myocardial perfusion imaging, myocardial fatty acid metabolism imaging, myocardial inflammation imaging, cardiac radionuclide angiography, and myocardial sympathetic nerve imaging [1]. The tracer ^123^I-MIBG is used to assess and visualize the activity of the sympathetic innervation in the heart. For decades, cardiac ^123^I-MIBG SPECT/CT examined various diseases [2,3].

Among these diseases, arrhythmogenic right ventricular cardiomyopathy (ARVC), a primary cardiomyopathy, leads to adverse right ventricle (RV) remodeling. This is triggered by the fibrofatty replacement of the myocardium [4]. As the disease progresses, the left ventricle (LV) is affected in three-quarters of patients [5,6]. Severe cardiac arrhythmias are one of the symptoms of ARVC and are attributed, among other things, to the loss of the sympathetic nervous system in the heart [7,8,9]. In ^123^I-MIBG scintigraphy, patients suffering from ARVC showed a lower uptake, especially in the RV [10,11].

Interestingly, the concept of neuronal degeneration is evident in other diseases, such as Parkinson’s disease (PD) and dementia with Lewy bodies (DLB). In addition to motor symptoms, PD also presents non-motor symptoms. One such non-motor appearance is autonomic dysfunction [12]. This occurs due to the impairment of the sympathetic nervous system, which can be evaluated by cardiac ^123^I-MIBG scintigraphy [13,14]. The DLB is reported to have sympathetic denervation, which can also be assessed with ^123^I-MIBG scintigraphy [14,15]. The cardiac ^123^I-MIBG-uptake in patients with DLB correlates with the degree of sympathetic denervation [16].

According to the biological definitions of PD and DLB, both involve α-synuclein pathology. Thus, they may be collectively considered α-synucleinopathies [17,18].

Previous studies detected the ^123^I-MIBG uptake as a ratio between cardiac and mediastinal uptake (heart-to-mediastinum-ratio, H/M-ratio), a semi-quantitative analysis [11,13,16]. For patients suffering from ARVC, an H/M ratio below 1.6 is reported as critical [11,19]. An H/M ratio below 1.77 in neurological patients is considered conspicuous for α-synucleinopathies [20]. Previous studies depicted the feasibility of standardized uptake value (SUV) as a quantitative parameter for SPECT/CT imaging [21,22,23] and examined the SUV of the LV and the RV in patients suffering from ARVC [24].

However, no head-to-head ^123^I-MIBG uptake analysis of the common pathophysiological process of denervation comparing different cardiac and neurologic diseases has been published yet. Therefore, this research project aimed to gain insight into cardiac ^123^I-MIBG imaging comparing cardiac and α-synucleinopathy patients sharing the pathophysiological mechanism of denervation.

We aimed to focus on the following aspects:

(I) Is it feasible to examine the RV and LV in ^123^I-MIBG SPECT of patients suffering from α-synucleinopathies to determine quantitative cardiac innervation (SUV)?

(II) We compared quantitative cardiac innervation (SUV) of patients with α-synucleinopathies with patients with definite ARVC.

(III) We compared semi-quantitative cardiac innervation (H/M-ratio) of patients with α-synucleinopathies and definite ARVC.

## 2. Materials and Methods

### 2.1. Study Cohort

We retrospectively analyzed patients who underwent cardiac ^123^I-MIBG scintigraphy from 2010 to 2022 at the Department of Nuclear Medicine, LMU University Hospital, LMU Munich, diagnosed with α-synucleinopathies or with definite ARVC. Diagnoses were obtained from the patients’ files of the Department of Neurology, LMU University Hospital, LMU Munich, and the Department of Cardiology, LMU University Hospital, LMU Munich.

Patients undergo clinical examination before and after the ^123^I-MIBG SPECT imaging. PD is diagnosed according to the Movement Disorder Society Clinical Diagnostic Criteria for Parkinson’s disease. The primary symptom is parkinsonism, defined as bradykinesia combined with rigidity or rest tremor, often accompanied by non-motor symptoms. In the second step, the underlying cause of parkinsonism is determined. Additional investigations are conducted to diagnose α-synucleinopathies [17,18,25]. The diagnosis of definite ARVC is made according to the modified task force criteria. They are divided into major and minor categories, including findings from echocardiography, magnetic resonance imaging (MRI), histology electrocardiography, and patient history [26].

Details on arrhythmias and initial diagnosis were gathered from the implantable cardioverter defibrillators (ICD) examinations and patient records. Arrhythmia was characterized by the occurrence of ICD intervention (e.g., antitachycardia pacing therapy, cardioversion, or defibrillation).

The local ethics committee has secured approval for this study (project number 22-0328).

### 2.2. SPECT/CT Imaging

Image acquisition was executed following a standardized protocol [11]. An average ^123^I-MIBG activity of 345.08 ± 79.27 MBq for definite ARVC patients and 194.91 ± 8.29 MBq for patients with α-synucleinopathies was applied. SPECT/CT was acquired using a low-energy, high-resolution collimator. For definite ARVC patients, the parameters were a 64 × 64 matrix, pixel spacing of 6.59, an energy window of 159 keV, a width of 15%, a zoom factor of 1.46, and a scan arc of 90°. The type of motion was step and shoot, with a frame duration of 30 s, and number of frames was 32. For patients with α-synucleinopathies, the parameters were a 128 × 128 matrix, pixel spacing of 4.42, an energy window of 159 keV, a width of 15%, a zoom factor of 1, and a scan arc of 180°. The type of motion was step and shoot, with a frame duration of 45 s, and number of frames was 32. In addition, a low-dose CT scan (130 kV, 20 mAs) was conducted. Image acquisition was performed 4 h after the tracer injection. All scans were performed with a Symbia TruePoint SPECT/CT scanner (Siemens Healthineers, Erlangen, Germany).

### 2.3. Image Analysis and Interpretation

Image reconstruction was executed using Hermia HybridRecon Software (Hermes Medical Solutions, Stockholm, Sweden, Version 4.0.6). The H/M ratio was measured as previously described [11].

The following analysis was performed using PCARD (PMOD Technologies LL, Fälland, Switzerland, Version 4.204). Using the SUV image calculation tool, image settings were changed to SUV by entering the injected dose of ^123^I-MIBG, time of application, height, and weight of the patient. The liver was used as the point of orientation for the fusion of SPECT and CT images.

**Figure 1:**
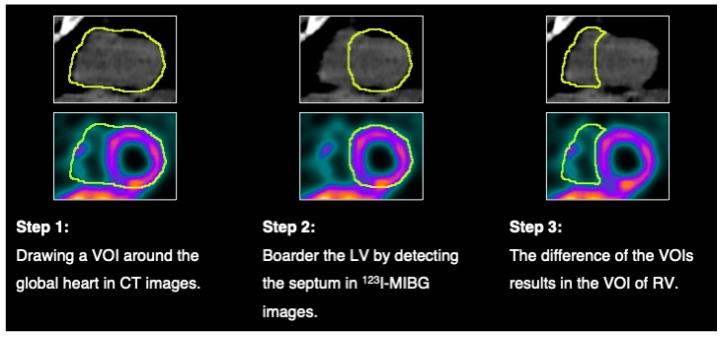
Image processing. The figure presents a patient with neurodegenerative disease.

Afterward, the images were reconstructed manually using the CT images, resulting in images of vertical long-axis (VLA), horizontal long-axis (HLA), and short-axis (SA). A volume of interest (VOI) was drawn across all heart slices in CT images. To determine the LV margin, the septum was detected in the CT and ^123^I-MIBG images, and the septum was assigned to the LV. The myocardium and the cavity were included. The result of VOIs of the global heart (heart), LV, and the RV is shown in Figure 1. SUV_median_, SUV_max_, and SUV_peak_ were determined in these VOIs. SUV_median_ was defined as the median value of the VOI pixel values, SUV_max_ was defined as the maximum pixel value, and SUV_peak_ was defined as an average of 1 mL placed in the VOI such that the average is maximized.

### 2.4. Statistical Analysis

For statistical analysis, RStudio (Version 2023.06.0+421) was utilized. Categorical or binary variables were expressed in absolute and relative frequencies. Metric variables were presented with their mean and standard deviation (SD). The data were tested for normal distribution using the Kolmogorov–Smirnov test. A *t*-test was performed to compare the two disease groups. Cohens’ d, receiver operating characteristic (ROC) curves, the area under the curve (AUC), and the DeLong test were used to compare the two methods. The Pearson correlation coefficient and the corresponding test were performed for correlation analyses. The level of significance was set at 0.05.

## 3. Results

Two groups resulted in patients suffering from definite ARVC (*N* = 20) and patients suffering from α-synucleinopathies (*N* = 8). α-synucleinopathies included patients with Parkinson’s disease (*N* = 5), and dementia with Lewy bodies (*N* = 3). The gender proportion (ARVC: 70% male, 30% female; α-synucleinopathies: 38% male, 62% female) and the weight of the patients (weight: ARVC: 79 ± 11 kg, α-synucleinopathies: 78 ± 7.5 kg) was similar across both groups. The age among the groups was different (ARVC: 46 ± 16 years, α-synucleinopathies: 68 ± 10 years). The duration between first diagnosis and ^123^I-MIBG imaging was 2.6 years (SD ± 3.5 years) for patients suffering from α-synucleinopathies and 6.6 years (SD ± 7.8 years) for patients suffering from ARVC. In patients with α-synucleinopathies, no arrhythmias were reported and were expected for one patient with sick sinus syndrome and atrial tachycardia, who received a pacemaker. Among patients with ARVC, no arrhythmias were reported in 7 patients, while arrhythmias were reported in 13 patients. The arrhythmias occurred on average 1117.15 days (SD ± 2120 days, median: 238 days) before ^123^I-MIBG imaging. Image processing and VOI assessment are illustrated in Figure 1.

### Comparison of α-Synucleinopathies and Definite ARVC

We compared SUV_median_, SUV_max_, SUV_peak_, and the H/M ratio of patients with α-synucleinopathies and definite ARVC.

**Figure 2:**
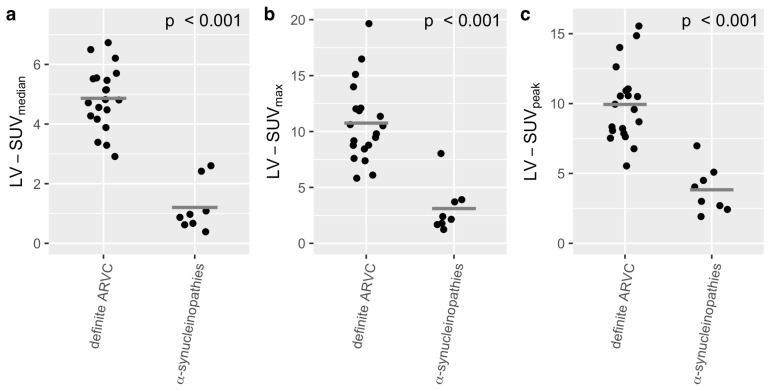
Comparison of the LV-SUV_median_ (**a**), LV-SUV_max_ (**b**), and LV-SUV_peak_ (**c**) between the ARVC group (*N* = 20) and the group of α-synucleinopathies (*N* = 8). The *t*-test showed significant differences in all categories. The gray crossbar represents the mean.

**Figure 3:**
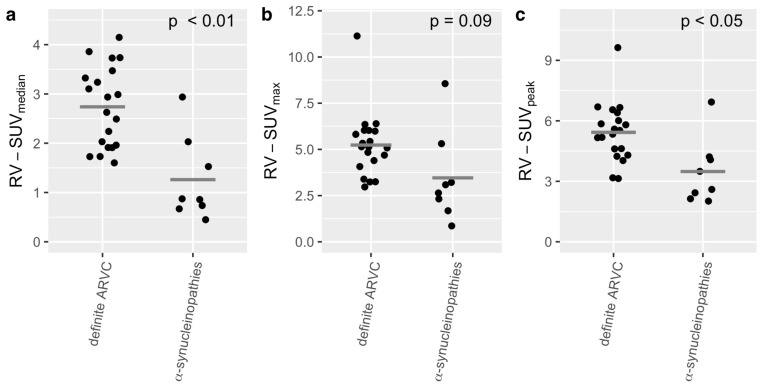
Comparison of the RV-SUV_median_ (**a**), RV-SUV_max_ (**b**), and RV-SUV_peak_ (**c**) between the ARVC group (*N* = 20) and the group of α-synucleinopathies (*N* = 8). The *t*-test showed significant differences in RV-SUV_median_ and RV-SUV_peak_ but not in RV-SUV_max_. The gray crossbar represents the mean.

**Figure 4:**
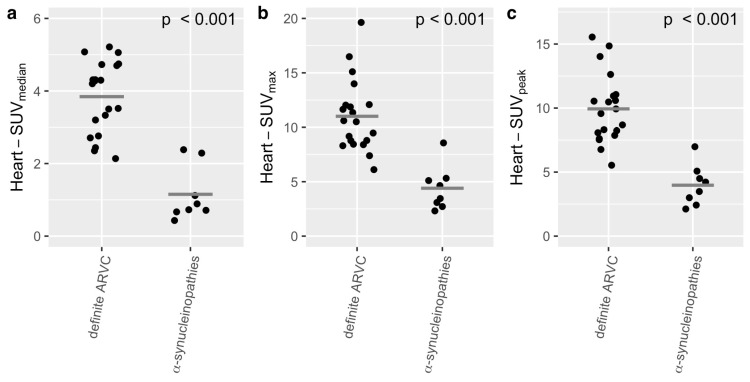
Comparison of the heart-SUV_median_ (**a**), heart-SUV_max_ (**b**), and heart-SUV_peak_ (**c**) between the ARVC (*N* = 20) group and the group of α-synucleinopathies (*N* = 8). The *t*-test showed significant differences in all categories. The gray crossbar represents the mean.

A comparison of the SUV_median_ showed a significant difference for LV-SUV_median_ (α-synucleinopathies: 1.2 ± 0.84, definite ARVC: 4.86 ± 1.04, *p* < 0.001, see Figure 2a), for RV-SUV_median_ (α-synucleinopathies: 1.26 ± 0.85, definite ARVC: 2.74 ± 0.82, *p* < 0.01, see Figure 3a), and for heart-SUV_median_ (α-synucleinopathies: 1.15 ± 0.76, definite ARVC group: 3.84 ± 0.99, *p* < 0.001, see Figure 4a).

A comparison of the SUV_max_ showed a significant difference for the LV-SUV_max_ (α-synucleinopathies: 3.11 ± 2.2, definite ARVC: 10.75 ± 3.49, *p* < 0.001, see Figure 2b) and heart-SUV_max_ (α-synucleinopathies: 4.40 ± 2.02, definite ARVC: 11.01 ± 3.31, *p* < 0.001, see Figure 4b). There was no significant difference for RV-SUV_max_ (α-synucleinopathies: 3.46 ± 2.44, definite ARVC: 5.24 ± 1.76, *p* = 0.09, see Figure 3b).

A comparison of the SUV_peak_ showed a significant difference for the LV-SUV_peak_ (α-synucleinopathies: 3.83 ± 1.68, definite ARVC: 9.94 ± 2.70, *p* < 0.001, see Figure 2c), for RV-SUV_peak_ (α-synucleinopathies: 3.48 ± 1.63, definite ARVC: 5.42 ± 1.45, *p* < 0.05, see Figure 3c), and for heart-SUV_peak_ (α-synucleinopathies: 3.98 ± 1.59, definite ARVC: 9.94 ± 2.70, *p* < 0.001, see Figure 4c).

**Figure 5:**
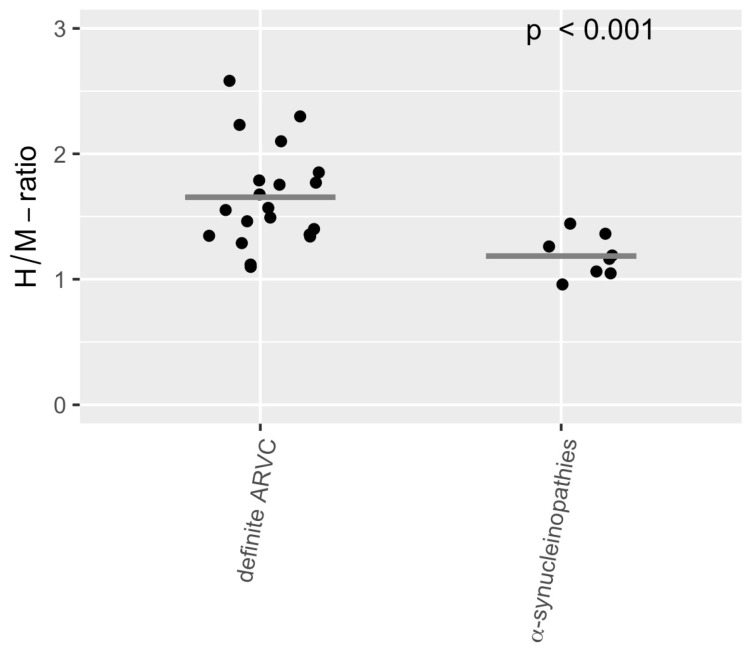
Comparison of the H/M ratio between the ARVC group (*N* = 20) and the group of α-synucleinopathies (*N* = 8). The *t*-test showed a significant difference. The gray crossbar represents the mean.

A comparison of the H/M ratio also showed significant differences (α-synucleinopathies: 1.19 ± 0.16, definite ARVC: 1.65 ± 0.4, *p* < 0.001, see Figure 5). Eleven patients with ARVC and all patients with α-synucleinopathies exhibited an H/M ratio below 1.6.

To compare heart-SUV_median_ and H/M ratio, Cohens’ d was calculated, indicating more significant group discrimination for SUV compared to H/M ratio (heart-SUV_median_: 2.88 (95%-CI: 1.71, 4.06), H/M ratio: 1.33 (95%-CI: 0.39, 2.27)). Heart-SUV_median_ and H/M ratio correlated strongly and significantly (r = 0.61, *p* < 0.001, see Figure S1).

## 4. Discussion

^123^I-MIBG imaging can be used not only to assess the severity of heart failure and arrhythmogenic diseases but also to investigate neurodegenerative diseases [1,14,27]. The objective of this study was to examine the difference between clinically diagnosed α-synucleinopathies and definite ARVC in cardiac ^123^I-MIBG SPECT. Furthermore, we investigated the difference between the semi-quantitative and quantitative assessments of ^123^I-MIBG images. It is debated whether a two-dimension assessment, such as the H/M ratio, is sufficient to evaluate a three-dimensional structure like the heart. Evidence suggests that a three-dimensional approach, based on SUV guided by CT-defined morphology, provides a more accurate assessment [28]. For this purpose, we evaluated the H/M ratio and SUV of cardiac ^123^I-MIBG images, examining the LV, the RV, and the global heart. It was feasible to differentiate between the LV and the RV in patients suffering from α-synucleinopathies. It has been shown previously that assessing the SUV of the LV in neurological patients is possible [29,30].

The direct comparison of H/M ratio and heart-SUV_median_ shows no significant preference for either of these methods. However, there is a slightly better AUC distinguishing between definite ARVC and α-synucleinopathies, although this difference is not substantial. Additionally, Cohen’s d indicates an advantage in SUVs. This aligns with a previous study demonstrating that the left ventricle-to-mediastinum-ratio correlates with the LV-SUV and the right-ventricle-mediastinum-ratio correlates with the RV-SUV in patients with cardiac diseases, such as ARVC [24]. Other studies found a correlation between H/M ratio and SUV_median_ in the LV among patients with cardiac or neurological diseases, e.g., PD and DLB [29,30].

In addition to current literature, our study shows that there is a significant difference between the quantitative SUV in patients with α-synucleinopathies and definite ARVC in all three territories, as well as a significant difference in the semi-quantitative H/M ratio. The only exception was the RV-SUV_max_, where the difference between the two diseases was not significant. We compared two diseases that are known for low cardiac uptake of ^123^I-MIBG [10,11,13,30]. There is a significantly lower cardiac uptake in patients with α-synucleinopathies than in patients with definite ARVC.

To illustrate the cardiac sympathetic nervous system, ^123^I-MIBG imaging is used in clinical practice, particularly in Japan and the United States. This is due, among other reasons, to the association between cardiac sympathetic nervous system dysfunction and severe cardiac complications [31]. For patients with ARVC, it is suspected that the impairment and subsequent decline of cardiac sympathetic nervous system is responsible for arrhythmias. Another reason for arrhythmias appears to be the pathology of ARVC, which leads in fibrofatty replacement of the myocardium and may contribute to cardiac electrical remodeling [4,32,33]. Severe arrhythmias until sudden cardiac death are frequently reported [7,8,9]. However, in patients with Parkinson’s disease and parkinsonism, arrhythmias and sudden cardiac death are rarely reported [34]. A low uptake of ^123^I-MIBG is associated with arrhythmias and sudden cardiac death in patients suffering from heart failure [35,36]. Jacobson et al. [19] reported a prognostic value of reduced ^123^I-MIBG uptake for cardiac events in patients with significant LV dysfunction. Nagahara et al. [37] reported that a reduced ^123^I-MIBG uptake in combination with LV function and plasma BNP level is a predictor for risk of sudden cardiac death. Verschure et al. [38] showed that the risk for ICD therapy increases in patients with an intermediate uptake decrease but is not as high in patients with very low uptake. Most patients with α-synucleinopathies exhibit cardiovascular dysfunction, but only a few show symptoms. α-synucleinopathies have been reported with orthostatic hypotension at varying frequencies. When orthostatic hypotension is present, there is also reduced cardiac sympathetic innervation; however, cardiac dysfunction is not the sole cause of hypotension [39,40]. Severe cardiac arrhythmias, however, are not reported or may be underrated. These results, combined with the observations of this study, suggest that there is a specific range of ^123^I-MIBG uptake levels, while both higher and lower uptake compared to each disease entity could help to detect the risk for arrhythmias. Interestingly, arrhythmias occur in ARVC patients frequently but not in patients suffering from neurodegenerative diseases, although the latter shows lower uptake. Potentially, disease-specific range values should be investigated and analyzed in future studies.

Although ^123^I-MIBG-SPECT is a standard method for studying the cardiac sympathetic nervous system, positron-emission-tomography (PET) offers several advantages, such as more detailed regional investigation of the myocardium, improved spatial and temporal resolution, and the ability to perform quantification. Regarding the latter, we could demonstrate that quantitative assessment is also feasible in ^123^I-MIBG imaging. A more detailed investigation of different regions within the denervated myocardium could enhance diagnostic accuracy in patients suffering from ARVC and α-synucleinopathies. Additionally, other tracers, such as ^11^C-catecholamine or ^18^F-LMI1195, are used in PET/CT imaging to investigate the cardiac sympathetic nervous system. While ^123^I-MIBG, ^11^C-catecholamine, and ^18^F-LMI1195 appear to have similar uptake patterns, they differ in their accumulation dynamics [41,42]. Further studies are necessary to explore their potential benefit in cardiac PET/CT imaging in patients with ARVC- and α-synucleinopathy.

### Limitations

As a limitation of this study, we cannot exclude the morphologic changes of the ventricles, e.g., dilatation, which might influence the SUV. Due to the retrospective character of the study, there is no follow-up for arrhythmias after imaging, so we could not correlate the occurrence of arrhythmia with the results of image analysis. Additionally, the ^123^I-MIBG scan parameters differed between the group of ARVC patients and those with α-synucleinopathies. Furthermore, the number of patients is limited, and there is no group of healthy individuals. Another limitation is that, due to the small sample size and the differing occurrence of the diseases, the two groups are not age- and gender-matched. For the interpretation of the onset of severe arrhythmias, it must be noted that two different reasons for arrhythmia in patients with ARVC are discussed: the decline of cardiac sympathetic nervous system and the fibrofatty remodeling of the myocardium.

## 5. Conclusions

We demonstrated that the analysis of the LV, RV, and the global heart in ^123^I-MIBG SPECT is feasible in patients suffering from α-synucleinopathies. ARVC and α-synucleinopathies exhibit a similar pathophysiological process, which is the denervation of the sympathetic nervous system. To our knowledge, this is the first study that compares cardiac ^123^I-MIBG images from patients with definite ARVC and patients with α-synucleinopathies, interestingly showing a significantly lower uptake in the latter. This difference is significant in both semi-quantitative and quantitative investigations. We did not observe a clear benefit in examining the LV or RV separately instead of the global heart or in using SUV_max_ or SUV_peak_ before SUV_median_. However, it appears that quantitative analysis has an advantage in distinguishing between ARVC and α-synucleinopathies. Furthermore, this might suggest that disease-specific SUV ranges should be applied instead of a normal joint range. In addition, our data might indicate that arrhythmias in the context of a decreased cardiac sympathetic nervous system are underestimated in patients suffering from α-synucleinopathies.

## Data Availability

The raw data supporting the conclusions of this article will be made available by the authors on request.

## Supplementary Materials

The following supporting information can be downloaded at: https://www.mdpi.com/article/10.3390/diagnostics15010024/s1, Figure S1: There is a strongly and significant correlation between the H/M-ratio and heart-SUV_median_ as tested by Pearson correlation (r = 0.61, *p* < 0.001); Figure S2: Receiver operating characteristic (ROC) curves and area under the curve (AUC) values were generated to compare heart-SUV_median_ (a) and H/M-ratio (b).
