# Supplementary material for: Diagnostic Efficacy of 123Iodo-Metaiodobenzylguanidine SPECT/CT in Cardiac vs. Neurological Diseases: A Comparative Study of Arrhythmogenic Right Ventricular Cardiomyopathy and α-Synucleinopathies
Source: Diagnostics (Basel). 2024 Dec 26;15(1):24. doi: 10.3390/diagnostics15010024 (PMC11720076; doi:10.3390/diagnostics15010024)
Supplement: Supplementary file 1 [file diagnostics-15-00024-s001.zip › diagnostics-3377516-supplementary.pdf]

Supplementary Data

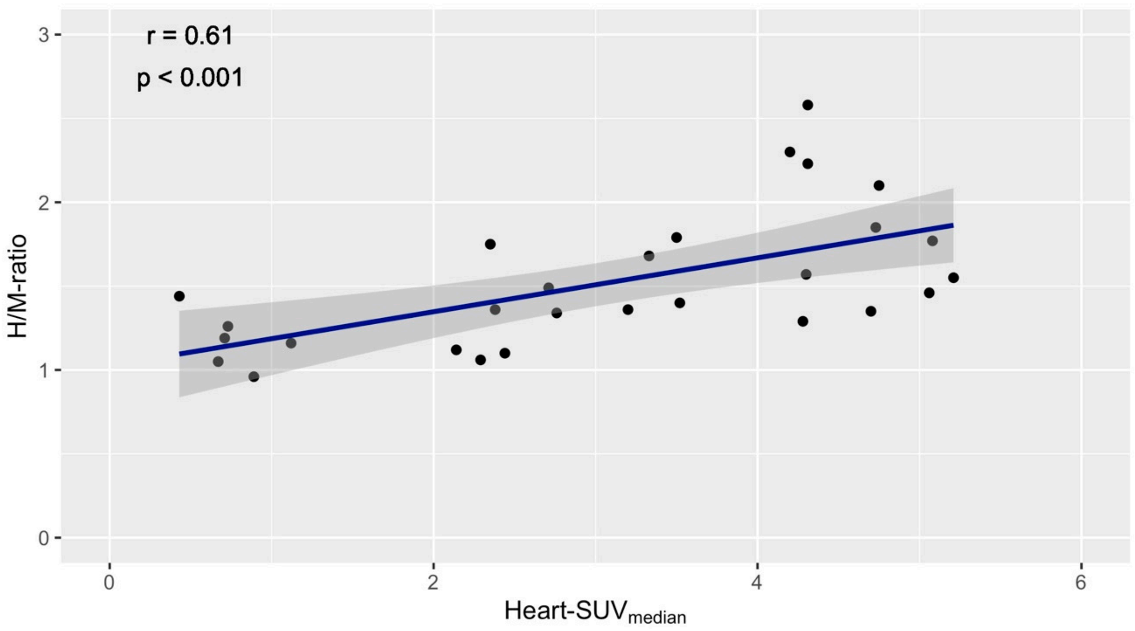

Figure S1

There is a strongly and significant correlation between the H/M-ratio and heart-SUV_{median} as tested by Pearson correlation ($r = 0.61$, $p < 0.001$).

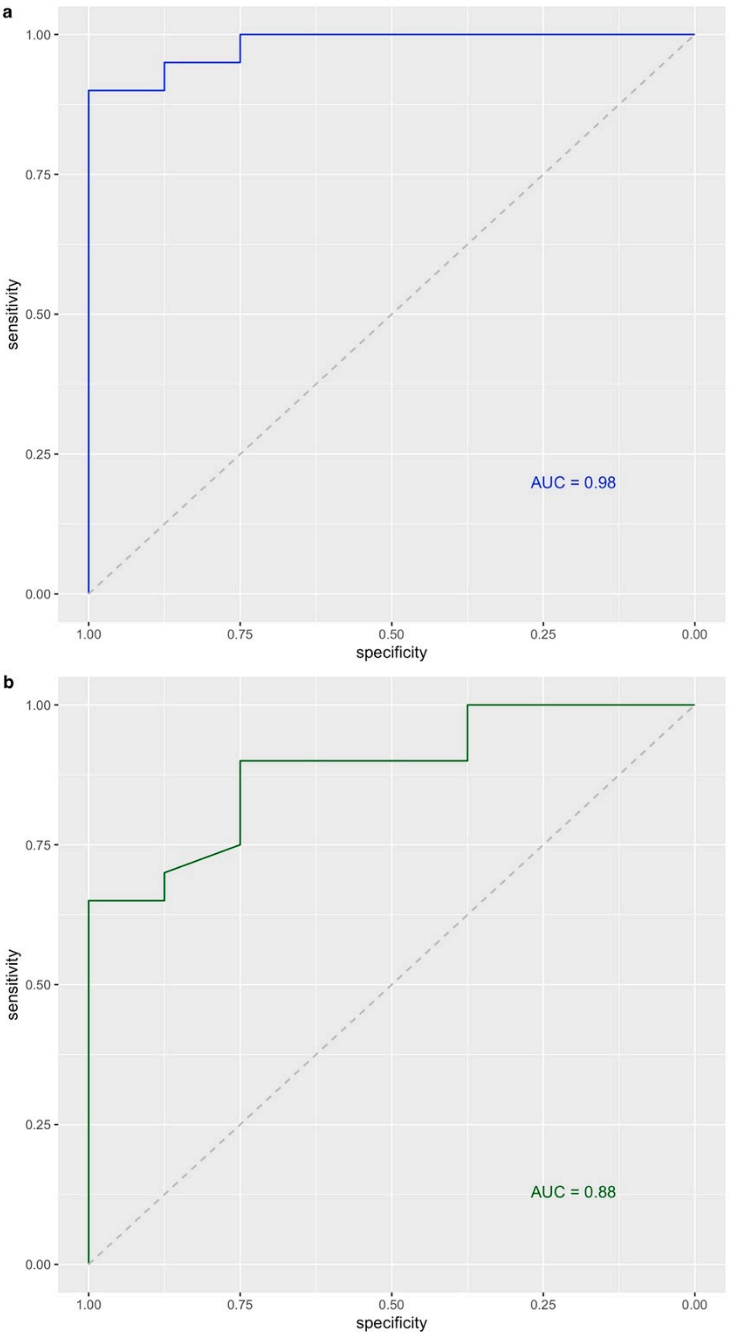

Figure S2

Receiver operating characteristic (ROC) curves and area under the curve (AUC) values were generated to compare heart-SUV_{median} (**a**) and H/M-ratio (**b**). The AUC for heart-SUV_{median} in distinguishing between definite ARVC and α-synucleinopathies was a slightly higher, but the difference was not statistically significant based on DeLong test ($p = 0.1$). Optimal cut-off-values to distinguish between definite ARVC and α-synucleinopathies were determined (heart-SUVmedian: 2.44 (sensitivity: 0.90, specificity: 1.00), H/M-ratio: 1.38 (sensitivity: 0.70, specificity: 0.89)). Even if there is no clinical advantage to this cut-off-values.
